# Account of Diseases in an Eastern District of London, from June 20 to July 20, 1804

**Published:** 1804-08-01

**Authors:** 


*Account of Diseases in an Eastern District of London, from June 20 to July 20, 1804.*

| | | | |
|---|---|---|---|
| ACUTE DISEASES. | | Convulsio | 1 |
| Peripneumonia | 2 | Ascites | 3 |
| Enteritis | 1 | Anasarca | 4 |
| Rheumatismus Acutus | 5 | Hydrothorax | 3 |
| CHRONIC DISEASES. | | Hæmorrhois | 4 |
| Tussis | 10 | Rheumatismus Chronicus | 14 |
| Dyspnœa | 5 | PUERPERAL DISEASES. | |
| Tussis cum Dyspnœa | 17 | Menorrhagia Lochialis | 6 |
| Hæmoptysis | 2 | Ephemera | 4 |
| Phthysis Pulmonalis | 4 | Abscessus Mammæ | 1 |
| Hepatitis Chronica | 2 | INFANTILE DISEASES. | |
| Scrophula | 3 | Pertussis | 7 |
| Chlorosis | 5 | Ophthalmia | 3 |
| Paralysis | 2 | Spina Bifida | 1 |
| Cephalalgia | 5 | Herpes | 5 |

The patient referred to in the list, as afflicted by convulsions, was frequently attacked by symptoms very nearly resembling those which sometimes precede the epileptic paroxysm. Epilepsia, indeed, in most instances, occurs so suddenly, as hardly to give any warning to the patient himself, or to those who are near him; but at other times the disease is preceded by some milder symptoms, and a giddiness, tinnitus aurium, pain of the head, palpitation of the heart, and slight spasmodic affections in different parts of the body, introduce the complete paroxysm. Several of these symptoms occurred in the case referred to. Considerable pain in the head usually accompanied the convulsive twitches in the face, and other spasmodic affections.

Being informed that medicines of the antispasmodic class had been very liberally administered, without procuring any relief, leeches were directed to be applied to the temples. From this evacuation considerable relief was obtained; and the bowels being kept open by gentle eccoprotics, the patient continued free from his complaint during a longer interval than he had before enjoyed.

Upon a return of the symptoms, leeches were again applied to the temples, and a blister was placed behind each ear, and kept open for a considerable time by ung. Sabin. since which he has not had any return of his complaint.

